# A 25.1% Efficient Stand‐Alone Solar Chloralkali Generator Employing a Microtracking Solar Concentrator

**DOI:** 10.1002/gch2.201700095

**Published:** 2017-11-29

**Authors:** Enrico Chinello, Miguel A. Modestino, Laurent Coulot, Mathieu Ackermann, Florian Gerlich, Demetri Psaltis, Christophe Moser

**Affiliations:** ^1^ School of Engineering ‐ STI Ecole Polytechnique Federale de Lausanne (EPFL) EPFL STI IMT LAPD BM4110 Station 17 CH 1015 Lausanne Switzerland; ^2^ Tandon School of Engineering New York University (NYU) Rogers Hall 600A Brooklyn NY 11201 USA; ^3^ Insolight Sarl Chemin de la Raye, 13 ‐ Ecublens CH 1015 Switzerland

**Keywords:** chloralkali, chlorine, hydrogen, multijunction photovoltaics, solar concentrators

## Abstract

Chlorine is a large‐scale chemical commodity produced via the chloralkali process, which involves the electrolysis of brine in a membrane‐based electrochemical reactor. The reaction is normally driven by grid electricity; nevertheless, the required combination of voltage–current can be guaranteed using renewable power (i.e., photovoltaic electricity). This study demonstrates an off‐grid solar‐powered chlorine generator that couples a novel planar solar concentrator, multijunction InGaP/GaAs/InGaAsNSb solar cells and an electrochemical cell fabricated via additive manufacturing. The planar solar concentrator consists of an array of seven custom injection‐molded lenses and uses microtracking to maintain a ± 40° wide angular acceptance. Triple‐junction solar cells provide the necessary potential (open‐circuit voltage, *V*_OC_ = 3.16 V) to drive the electrochemical reactions taking place at a De Nora DSA insoluble anode and a nickel cathode. This chloralkali generator is tested under real atmospheric conditions and operated at a record 25.1% solar‐to‐chemical conversion efficiency (SCE). The device represents the proof‐of‐principle of a new generation stand‐alone chlorine production system for off‐grid utilization in remote and inaccessible locations.

## 1 Introduction

Photovoltaics (PV) technologies that can turn solar energy into useful electricity have surged in the last decades, and their utilization is ever increasing.^1^ The development of solar technologies has paved the way toward the robust and efficient off‐grid operation in remote areas where the grid is inaccessible or where extending the power connection lines would not be economically justifiable. Besides its direct use for lighting, appliances, etc., stand‐alone photovoltaic electricity can be utilized to generate a valuable chemical feedstock via electrochemical processes. They are the natural entry point for PV integration, as these processes specifically require direct electricity to operate. Their scalability, coupled with the possibility to modulate the load and possibly operate on‐demand, facilitates the implementation of time‐varying renewable energy sources.^2^ Most of the research efforts have been recently devoted to the investigation of solar hydrogen devices, following two distinguished paths: photoelectrochemical (PEC) or photovoltaic–electrolysis (PV–E) devices. The latter approach appears to be more technologically mature for short‐term implementation and have been demonstrated using multijunction solar cells^3^ and inexpensive silicon cells.^4^ While most efforts in the past decades have focus on the development of cost‐effective hydrogen‐generators, little attention has been dedicated to the development of stand‐alone solar‐reactors for the electrochemical generation of different commodities, e.g., halides that can already count on an established industrial practice.^5^

Chlorine is used as a feedstock for the manufacture of numerous products in more than 50% of all the industrial processes.^6^ The main route to produce chlorine is the chloralkali process, one of the largest electrochemical operations in industry; it accounts for a yearly energy consumption of 150 TWh, 1% of the global overall and 2% of the US electrical production.^7^ Molecular hydrogen and hydroxide ions (which can later be extracted as sodium hydroxide) are generated in the cathodic reaction. The predominant chloralkali electrochemical reactors implement membranes^8^ that separate a De Nora DSA anode and a nickel‐based cathode.^9^ The anodic coating is a metallic oxide blend, constituted of a mixture of ruthenium and titanium oxides mainly. Beside the importance of chlorine and sodium hydroxide as chemical feedstocks, hydrogen could be fully recovered, power to a significant extent the process itself or serve as an energy vector and further impact different sectors (e.g., mobility). It is therefore evident that the chloralkali process (and oxidation of halides in general) holds an intrinsic economic advantage when compared to the traditional water‐splitting reaction; the oxidation of chloride, which is valuable (price ranges between USD 230 to 500 ton^−1^ 5, 10), can replace water oxidation and substitute a product often discarded such as oxygen. This operation permits us to obtain three valuable products, molecular hydrogen and sodium hydroxide at the cathode and molecular chlorine at the anode, thus maximizing the economical profitability of the electrolysis.

High efficiency solar technologies are favored when employed in solar chemical processes as they restrain nonmodule depending costs, capital investments, and land utilization.^11^ Moreover, as independent stand‐alone devices suffer from the intermittency of the solar illumination, high‐efficiency is critical in order to maximize the chemical production when the source is available and allow the feedstock utilization as continuously as possible. Silicon‐based modules account for the dominating share in the PV market.^12^ Several demonstrations have attempted to deploy silicon solar cells to produce chlorine; these studies considered commercially available modules to produce sanitizing agents for water disinfection at relatively low efficiencies (estimated <10%).^13^, ^14^, ^15^, ^16^ Other reports have highlighted the effect of chlorine and chloride radicals generation for silicon PV‐powered wastewater treatment utilities.^17^, ^18^, ^19^ Nevertheless, these demonstrations did not represent a practical implementation of the chloralkali process and chlorine was utilized within the reaction chamber and could not possibly be extracted and stored. Multijunction gallium arsenide (GaAs)‐based solar cells can provide a sufficient working potential avoiding series connection^20^, ^21^ and are therefore better suited for high‐efficiency solar chloralkali applications. Due to the high cost of materials, substrates and processes, their economic viability is limited, and the implementation of solar concentrators is desirable to reduce the size of the GaAs cells used. A significant amount of work has been devoted to the development of cost‐effective concentrated GaAs modules which has led to a progressive decrease in their cost.^22^, ^23^ This drop has relaxed the constraints imposed to the solar concentrators in order for the operation to be economically justifiable,^24^ allowing alternative optical designs to enter the field.^25^

**Figure 1:**
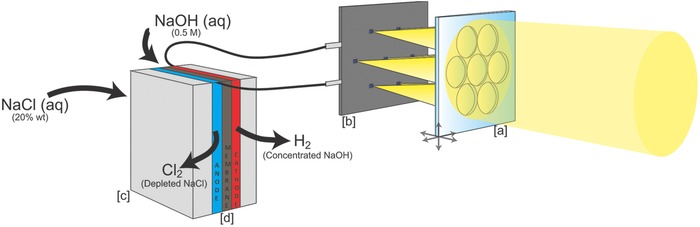
Solar chloralkali device overview. a) Optical planar concentrator, lenses array. b) Triple‐junction GaAs‐based solar cells mounted on PCB. c) Electrolyzer 3D printed metallic flow plates. d) Electrochemical cell, composed by a nickel cathode and a DSA insoluble anode, separated by cation exchange membrane.

In this study, we present a small‐scale solar powered chloralkali generator that employs multijunction InGaP/GaAs/InGaAsNSb solar cells, a 5 cm^2^ novel planar optical concentrator that tracks the sun throughout the year with minimal displacement requirements and a custom‐made electrochemical reactor fabricated via additive manufacturing. Thorough components design ensures that the output of the PV cells directly drives the brine electrolysis process with an unprecedented 25.1% solar‐to‐chemical energy conversion efficiency (SCE) under natural sunlight (**Figure** **1**). The high conversion yields are achieved modeling, sizing and fabricating the device components targeting the current‐load match; this also ensures stability of operation over a broad range of possible working conditions.^26^ To the best of our knowledge, this is the first study reporting (i) practical conversion efficiencies as high as 25% under real atmospheric conditions, (ii) the deployment of our novel planar concentrator to illuminate multijunction solar cells, (iii) a compact system (<5 cm^2^) than can condition up to 6 L of water per day (enough for the needs of 1–2 persons in an underdeveloped country).^27^

This lab scale prototype is designed so that it can be readily scaled to m^2^ panels.^28^ Substituting a relevant share of grid electricity consumption, our approach can serve as a vector toward a significant decarbonization of the chloralkali industry, that would benefit from independent and lower‐cost operation exploiting solar electricity during the day.^29^ More significantly, this system demonstrates the technological proof of principle of a potentially portable, independent solar powered chloralkali generator that can be implemented for chlorine production in off‐grid rural and remote locations; our platform could therefore serve not only to produce valuable commodities but above all to provide an effective chemical agent for water treatment purposes.

## 2 Results and Discussion

**Figure 2:**
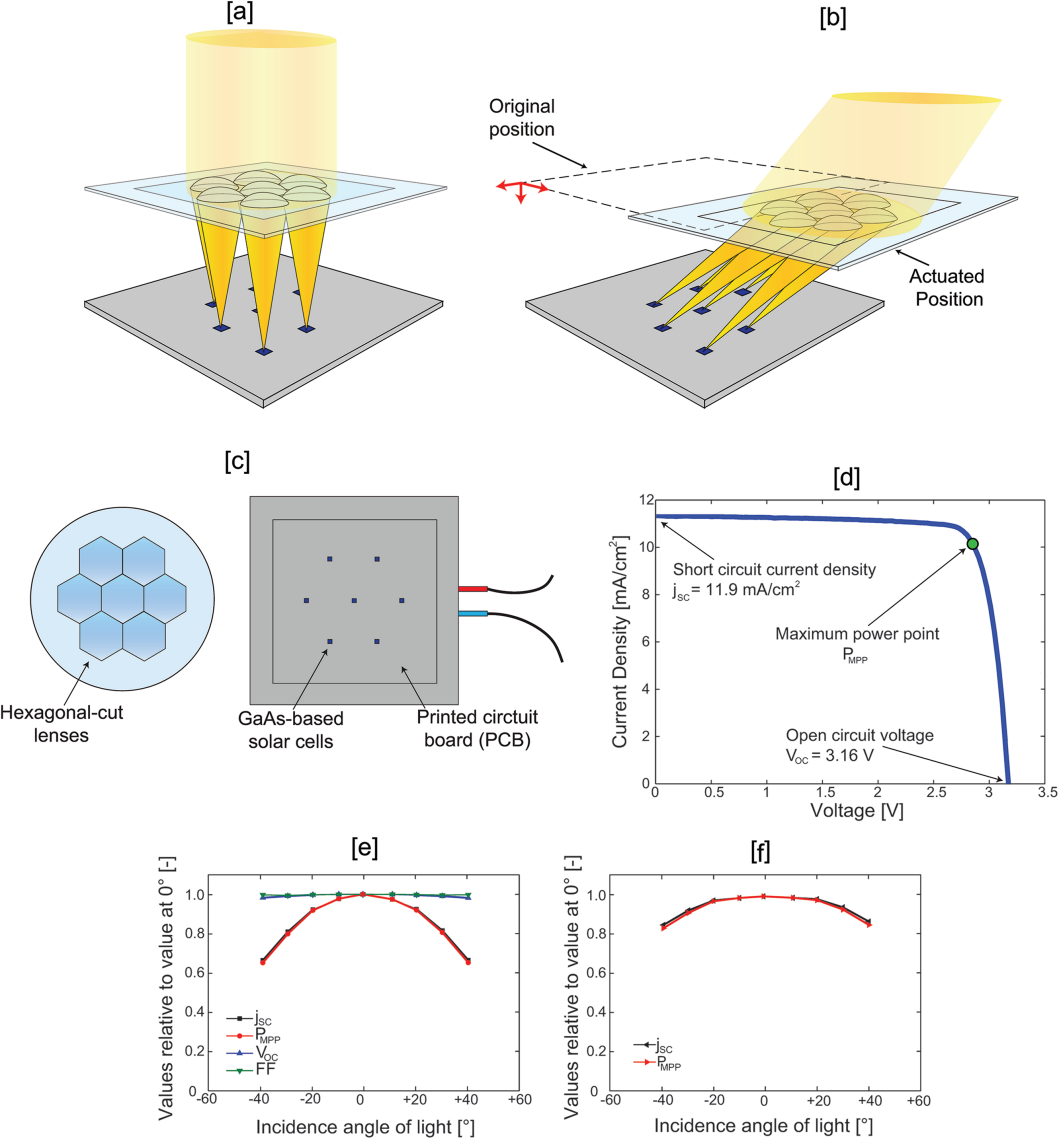
Insolight solar concentrator. a) Normal incidence case—optical efficiency is 100%. b) Intermediate incidence tilt. c) Assembly certified by Fraunhofer ISE. d) Certified polarization curve (direct normal irradiance 883 W m^−2^). e) Electrical performance of the assembly concentrator‐PV for different illumination angles, certified by Fraunhofer ISE. f) Electrical performance of the assembly concentrator‐PV corrected for the effective aperture area, certified by Fraunhofer ISE.

The solar powered chloralkali device employed three main components: multijunction GaAs‐based photovoltaic cells, an optical planar concentrator and a 3D printed electrochemical cell. The photovoltaic (PV) cells were illuminated using a novel planar solar concentrator, whose development was carried out at Insolight Sarl (Ecublens, Switzerland). We obtained lenses capable of guaranteeing solar tracking with ±5 mm displacements in the x–y–z space; the system eliminates the need to add the complex rotational mechanics typically employed in concentration photovoltaics.^30^ The main advantages of our optical planar concentrator are: (i) assembly in flat frames similar to those commonly used by silicon modules manufacturers thanks to the system planarity and the minimal lateral displacements needed; (ii) single lens design gives superior performance in terms of optical losses (estimated at 8%) compared with two lenses design frequently used for planar concentrators^31^, ^32^; (iii) scalability of the demonstrator to m^2^‐scale panels (**Figure** **2**a,b).

A seven lenses array prototype with input aperture 4.809 cm^2^, arranged in a hexagonal configuration was certified by Fraunhofer ISE (Freiburg, Germany) together with the solar cells (Figure 2c). Under real atmospheric conditions, direct normal irradiance (DNI) 883 W m^−2^ and air temperature 16.6 °C, the assembly concentrator cells recorded a short‐circuit current density (*j*_SC_) of 11.9 mA cm^−2^, an open‐circuit voltage of 3.162 V and an 85.6% fill‐factor (FF) (Figure 2d). The computed solar‐to‐electricity efficiency was 36.4%. Indoor testing was performed to measure the solar concentrator optical efficiency as a function of the input illumination angle. Experiments showed *j*_SC_ and power at maximum power point (PMPP) decreasing from 100% to 80% when tilting the input beam from 0° (normal direction) to 40° (measured from the normal axis) (Figure 2e,f).

The chloralkali reaction, described in Equation (1), occurred within a 3D printed electrolyzer. This gave us the possibility to exploit total design freedom and to rapidly test our prototypes

(1) $$2\mathrm{NaCl}\;+\;2H_{2}O\;\to \;{\mathrm{Cl}}_{2}\;+\;H_{2}\;+\;2\mathrm{NaOH}\;E^{0}\;=-2.1860\;V\;\;(\mathrm{SHE})$$

The industrial chloralkali process employs a working voltage within 2.4–2.7 V, with current densities within 1.5–7 kA m^−2^ at the optimal working temperature 70–90 °C; the NaCl concentration in the brine anolyte is 20% wt, whereas the NaOH content of the catholyte is typically 32% wt.^33^, ^34^

State‐of‐the‐art DSA anode and Nickel‐based activated cathode were employed to perform the anodic and cathodic reaction (Equations (2) and (3), respectively)

(2) $$2{\mathrm{Cl}}^{-}\;\to \;{\mathrm{Cl}}_{2}\;+\;2e^{-}\;E^{0}\;=\;1.3583\;V\;\;(\mathrm{SHE})$$

(3) $$2H_{2}O\;+\;2e^{-}\;\to \;H_{2}\;+\;2{\mathrm{OH}}^{-}\;E^{0}\;=\;-0.8277\;V\;\;(\mathrm{SHE})$$

**Figure 3:**
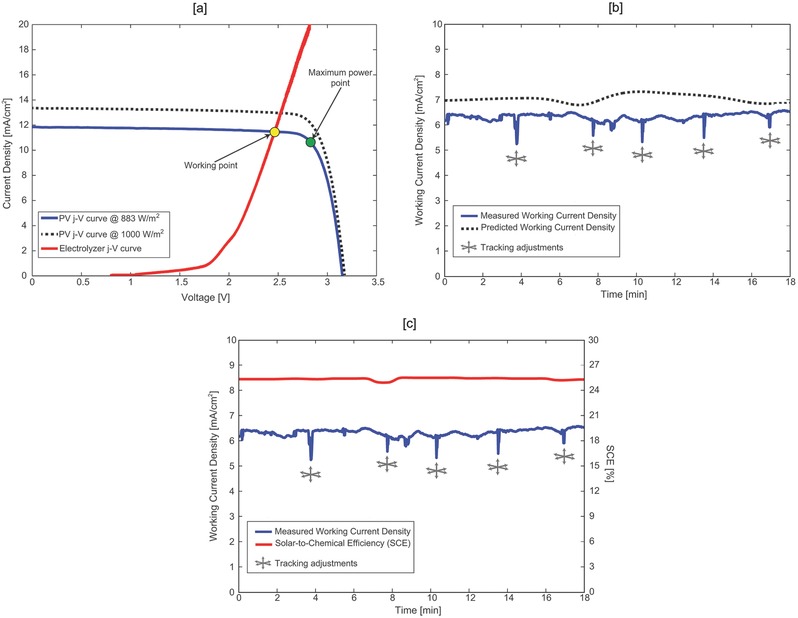
a) Chloralkali device working point. Blue line and black dashed line depict the PV polarization curve certified by Fraunhofer ISE (DNI 883 W m^−2^) and extrapolated at DNI 1000 W m^−2^. The red line shows the polarization curve for the electrolyzer. b) Device operating current (6th January 2017, 12:15‐12:35). The blue line depicts the recorded current through the device, the black dashed line represents to the computed operating current (at 2.7 V); the gray symbols correspond to the intervals in which the tracking was activated. c) Device operating current (blue) and correspondent solar‐to‐chemical conversion efficiency (SCE—red). It has to be noted that the surface reference for current densities calculations is the input aperture of the solar concentrator (i.e., 4.809 cm^2^); the brine electrolysis load (red curve in (a)) has therefore to be scaled to the effective electrode area in order to assess the electrolyzer performance.

In order to guarantee high efficiency and stability of operation throughout the different operative conditions, the device must be operated at a voltage lower that the solar cells maximum power point voltage (*V*_MPP_). This condition prevents conversion yields from dropping as the electrolyzer operation degrades.^26^ An effective component dimensioning targeting the load‐matching ensures the fulfillment of this condition (**Figure** **3**a).

The complete chloralkali device was tested under outdoor atmospheric conditions at the EPFL campus in Lausanne, Switzerland (coordinates: 46.521° N; 6.565° E). Solar displacements were tracked throughout the experiment via three‐axis linear millimetric movements of the lenses array, driven by three linear stage electric motors. Figure 3b reports the current circulating through the entire prototype while being exposed to natural sunlight (blue line). The tracking stages were activated manually for fine adjustments five times during the outdoor experiment (grey symbols). Solar irradiance data were subsequently retrieved from the Solar Energy and Building Physics Laboratory (LESO‐LB, EPFL) online database (http://leso2.epfl.ch/eibknx/index.php); DNI values within 537–615 W m^−2^ were calculated based on the direct horizontal irradiance (DHI) and the angle of incidence (AOI). The expected working current at the voltage 2.7 V was thus calculated using DNIs and the *I*–*V* curve Fraunhofer ISE certified (formula is reported in Table S1 in the Supporting Information). The results are depicted in (Figure 3b—dashed black line). The results show good agreement between the recorded and the projected values over the entire measurement timeframe (≈20 min). Discrepancies up to 10% were observed and attributed to the small location difference between the weather station and measurement site.

The SCE of the system was calculated considering the standard cell working potential associated with the total Gibbs free energy variation in standard conditions for the simultaneous evolution of Cl_2_, H_2,_ and NaOH

(4) $$\mathrm{SCE}=\frac{j\cdot E_{\mathrm{cell}}^{0}\cdot \eta_{\mathrm{Faraday}}}{P_{\mathrm{solar}}}$$

*j* being the working current density circulating in the device [mA cm^−2^]; $E_{\mathrm{cell}}^{0}$ is the standard electrochemical working cell potential, 2.188 V; η_Faraday_ is the faradaic efficiency of the electrochemical process; *P*_solar_ the solar irradiance power (i.e. DNI values retrieved from the weather station) [mW cm^−2^].

The calculation considered the contribution of all the reaction products, Cl_2_, H_2_, NaOH. Faradaic efficiencies were demonstrated to be 96%–99% (Figure S2, Supporting Information); the nonideality derives from the main anodic competitive reaction, O_2_ evolution. The common industrial practice reported in literature suggests an oxygen content of 1.5%–2% with no anolyte acidification.^35^

Overall SCE yields within 24.7–25.1% were observed (Figure 3c; Figure S3, Supporting Information). To the best of our knowledge, these represent the highest reported efficiency values for a solar brine splitting device. Throughout the experiment, H_2_ and Cl_2_ production rates were determined to 1.09 mg h^−1^ and 38.6 mg h^−1^, respectively); additional details are reported in Figure S3 (Supporting Information). The voltage efficiency of our electrochemical reaction was estimated to be ≈70%, which is consistent to those of commercially deployed systems.^34^, ^36^

Figure 3b demonstrates the capability of our solar powered chloralkali device to produce two valuable products, molecular chlorine and molecular hydrogen, at unprecedented efficiencies using natural sunlight. Our simple microtracking mechanism, based on the advanced optical design of our solar concentrator, was periodically activated to cover the solar disk displacements and maintain solar to product efficiency as high as 25.1%.

**Figure 4:**
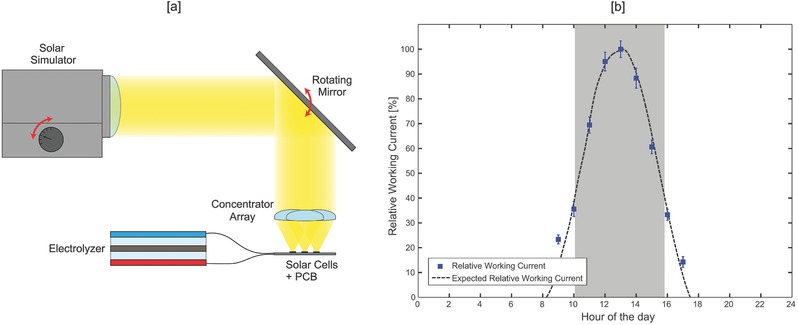
a) Experimental setup for daily tracking reconstruction. Solar simulator power and beam direction are adjusted to recreate solar intensity and angle for each hour of the day. b) Relative comparison of measured and computed values (blue dots and dashed black line, respectively); darker gray region shows the ±40° solar incidence angle.

In order to assess the performance of the solar chloralkali device under the illumination conditions of a typical summer day, we characterized the system under indoor simulated sunlight. The Illumination intensity and direction were reconstructed by tuning the solar simulator beam power and incidence angle, in order to recreate solar DNI and incidence direction of each hour of the day (**Figure** **4**a). Experiments were carried out proceeding by datapoints (i.e., hours of the summer day), illuminating the PV cells for 3 min for each set of illumination intensity and angle, averaging the mean working current and calculating the standard deviation—details in Figure S4 (Supporting Information) and Table S1 (Supporting Information). Results were then compared to the predicted values, based on weather data obtained from EnergyPlus database (https://energyplus.net/weather—U.S. DOE) and optical efficiencies derived from the Fraunhofer ISE certification. Outcomes are reported in (Figure 4b) showing measured (blue dots) and computed currents (dashed black line). The error bars correspond to the standard deviations of the 180 s measurement for each point. Values are plotted in relative terms due to the inability of the Xe‐lamp solar simulator to fulfill the spectral match required for multijunction cells—spectrum is reported in Figure S5 (Supporting Information)—and therefore recreate effectively natural sunlight conditions. The scarcity of power in the UV‐blue and infrared regions prevented the reliable utilization of Xe‐lamp simulators to illuminate GaAs cells and the problem has already been highlighted in the PV community.^37^, ^38^, ^39^ Current densities were normalized with respect to the maximum value (i.e., corresponding to 13 h conditions, 3.6 mA cm^−2^). Oriented toward the zenith direction, the concentrator was capable of illuminating the solar cells for each hour of the day (9 AM–5 PM) via millimetric motions in the x–y–z space of the concentrator optics. Figure 4b shows excellent agreement between the measured and the predicted values and demonstrates the extensive angular range of operation of our solar chloralkali device. The results demonstrate the effective use of the system throughout an entire day (Table S1, Supporting Information). Calculations based on irradiance data demonstrated that the device could capture up to 65% of the yearly solar power when exposed according to the local optimal tilt; in those conditions, the value is roughly location independent. Given the micromovements of the concentrator optics, the power consumption associated with tracking is negligible.

The stability of the solar chloralkali generator was tested under simulated sunlight in the most severe working conditions of the reconstructed summer day (i.e., corresponding to the 13 h data point). The device demonstrated its capability to operate continuously at high efficiency for more than 100 h with standard deviation from the mean value of ≈1.4%—Figure S6 (Supporting Information).

Calculations based on meteorological datasets predicted the ability of our 5 cm^2^ independent device to generate ≈43.8 g year^−1^ of Cl_2_ in Lausanne (Switzerland), exceeding of 114 g year^−1^ in higher irradiance locations (e.g., Phoenix, U.S.). Those annual productions are capable of sanitizing organic contaminants contained in 870 and 2280 L of water (2.4 and 6.2 L d^−1^—50 mgCl_2_ L^−1^), respectively; volumes reduce to 146 and 380 L (0.4 and 1.1 L d^−1^) in case of a heavily contaminated effluent (300 mgCl_2_ L^−1^).^40^

A preliminary technoeconomic study was conducted in order to assess the production cost of chlorine via solar‐powered routes. Calculations took into account similar devices to that demonstrated experimentally, comprising an optical concentrator, GaAs‐based solar cells and commercially available electrocatalysts in a membrane‐based reactor. The devices were dimensioned in order to generate 2.5 kg of Cl_2_ per day. This was estimated to be sufficient for conditioning a daily volume of 50 000 L of water with 50 mgCl_2_ L^−1^, satisfying the clean water needs of a small‐size hospital (40 patient beds, 200 000–500 000 square feet) in a developed country.^41^ Two locations were considered in the computations, Lausanne (CH) and Phoenix (U.S.). Capacity factors were calculated as 0.08 and 0.21, respectively; this reflected on a smaller active area required in Phoenix rather than Lausanne (4 and 10 m^2^, respectively). It has to be noted that all calculations were referred to the solar concentrator input aperture surface. Levelized costs of solar chlorine (LC_Cl2_) were calculated for a 20 years device lifetime, considering the replacement of Nafion membranes every 5 years^24^ and electrocatalysts lifetime of 10 years, although DSAs have demonstrated to operate for longer life spans.^34^, ^36^ These assumptions were intended to produce realistic outcomes, given the complexity and the volatility of a technoeconomic analysis.

LC_Cl2_ were calculated to be 400–680 and 150–250 $ ton^−1^ in Lausanne and Phoenix, respectively. The results depended on the optical concentrator and GaAs semiconductor considered cost. Results in Lausanne were penalized by the lack of direct radiation for a greater share of the year, which resulted in a lower capacity factor and therefore in a larger active surface to be employed. Nevertheless, the LC_Cl2_ for both locations indicated that solar‐powered devices are a competitive route to generate chlorine.^5^ Additionally, the current average market price could be beaten in high‐irradiance locations (e.g., Phoenix) by switching from grid‐fed to solar‐powered reactors.

The calculation details can be found in Table S1 (Supporting Information) and Figure S7 (Supporting Information).

## 3 Conclusions

This study demonstrates a solar chloralkali device composed of a planar solar concentrator, triple‐junction GaAs‐based solar cells and a custom‐built electrochemical reactor fabricated via additive manufacturing (3D printing). The novel solar concentrator used a millimetric triaxis movement to follow the solar disk over a large angular range (±40°). These allowed for the stable solar chlorine generation under natural sunlight condition, with efficiencies reaching an unprecedented 25.1% SCE. The record conversion efficiency was achieved via thorough design in order to ensure optimal load‐matching in a broad range of working conditions (i.e., as the electrolyzer performance degrades); this allowed to more than double conversion yields previously reported.^13^, ^14^, ^15^, ^16^ Furthermore, the device demonstrated has the ability to track the sun throughout prolonged periods of illuminations, such as the one of the typical summer day reconstructed indoor. Replacing grid‐powered with solar‐powered reactors bears economic benefits as well; the possibility of reducing the current chlorine market price up to 34% was demonstrated.

This demonstration represents a new‐generation functional proof‐of‐principle of a stand‐alone solar chloralkali generator. The solar‐electrolysis technology platform we have developed and demonstrated carries the potential to foster renewable energy sources penetration in the chloralkali industry, contributing to a significant reduction of its CO_2_ emissions associated with grid electricity consumption. Furthermore, an independent and potentially portable solution such as the one proposed is particularly suitable for implementation in rural or remote locations, where the electric grid is either inaccessible or impractical but sunlight is plentiful. Chlorine can be employed in a variety of applications including emerging solutions for water sanitization in isolated locations, where water effluents are generally contaminated with microorganisms bearing severe diseases. The unprecedented high efficiency presented is crucial under these circumstances in order to mitigate the reliance on an intermittent source as solar power, maximizing the chemical production. Theoretical simulations have demonstrated the capability of our 5 cm^2^ solar‐chlorine device to sanitize significant water volumes even under severe circumstances (yearly average: 0.25–4 L d^−1^, depending on the treatment severity). Upscaling of our compact prototype could address the sewage issues of large communities, where chlorine can be generated efficiently on‐site and at a lower cost, avoiding expenses and concerns associated with chlorine transportation.

Future studies should focus on the recovery and utilization of the hydrogen byproduct, which can be valorized as an energy carrier for direct electricity generation, transportation applications or to supplement the energy used to drive the chloralkali process.

## 4 Experimental Section

*Solar Concentrator*: The prototype used seven 0.6 × 0.6 mm^2^ triple‐junction InGaP/GaAs/InGaAsNSb (bandgaps 1.9/1.4/1.0 eV) solar cells in parallel. The single lens, radially symmetric and biconvex, was designed using an optical simulation tool to target the maximum achievable flattening of the Petzval field curvature for a wide angular input range. The solar concentrator consists of a planar hexagonal array of custom shaped lenses, fabricated in moldable silicone (MS‐1002) injected in an aluminum mold. In normal incidence conditions, the focal length was ≈6 mm. Our solar concentrator can illuminate multijunction GaAs‐based solar cells with a concentration ratio ≈180× over a wide range of beam incidence angles (±40° certified with optical efficiencies >80%).

*Electrolyzer and Electrolyzes*: Commercial electrode sheets were purchased from Industrie De Nora S.p.A. (Milan, Italy). A titanium plate (1.5 mm thick) coated with a MMO blend (DSA) was utilized to carry out the chloride ion oxidation, Equation (2). The cathodic reaction, Equation (3), occurred on the activated surface of a nickel grid (0.5 mm thick). Both electrodes were laser‐cut in a circular shape, Ø 1.3 cm, active area 1.3 cm^2^. Two identical flow plates were designed to distribute the electrolytes and hold the electrodes; they were 3D printed in steel, electroplated with gold and polished by Shapeways Inc. (New York, USA). The material choice ensured high electrical conductivity and robustness of the conductive layer over chlorine evolution and a corrosive electrolyte. Two additional pieces were 3D printed in VeroWhite using an additive manufacturing Stratasys tool (Object500 Connex); these provided casing for the electrodes and the membrane. Nitrile rubber (NBR) 70° O‐rings were utilized to seal the device.

The membrane material employed was DuPont Nafion 117, 183 mm thick. An exploded view of the overall assembly is provided in Figure S9 (Supporting Information). The anolyte solution was a 20% wt NaCl solution, prepared by dissolving highly pure salt (>99.5%), purchased from Roth GmbH, in deionized water. It was acidified to ensure selectivity of molecular chlorine over hypochlorite and chlorate by adding hydrochloric acid (Merck Millipore, ≥37%) to pH ≈ 1.5^36^ (monitored with a VWR pH110 pH‐meter, calibrated using Merck Certipur buffer solutions—pH 4.01/7.00/10.01). The catholyte solution was a 0.5 m NaOH solution (2% wt), prepared using pure caustic soda purchased from Reactolab SA. The catholyte concentration was reduced with respect to the industrial practice reference due to the moderate currents circulating in the electrolyzer. Both solutions were circulated via peristaltic pumps (New Era Pump Systems Inc. NE‐9000) at a working flow rate 20 mL min^−1^. Anolyte and catholyte were not replenished, given the little fraction of ionic content converted in the experiments. Two hotplates (VWR International VMS C‐7) heated the solutions, monitoring the temperature using two 10″ steel temperature probes (VWR International) immersed in the bulk solution under continuous stirring. The solutions temperature set point was 80 °C. Since the reaction kinetics degrades as the temperature decrease, it is worth noting that a stand‐alone device should comprise a solar heater. All electrochemical experiments were carried out utilizing a Bio‐logic potentiostat VSP‐300 or a Keithley 2401 source‐meter, at 20 mV s^−1^ scan rate.

*Outdoor Testing*: The solar chloralkali device was tested under real solar illumination on 6th January 2017, at the EPFL campus in Lausanne. Initially, the optical concentrator input aperture was aligned to the local zenith position, elevation angle 25.05°, azimuth angle 180°. Measurement was performed between 12:15 and 12:35, activating the three linear trackers five times to maintain excellent alignment. Solar irradiance data were obtained from the Solar Energy and Building Physics Laboratory (LESO‐LB, EPFL) online database. Total and diffuse horizontal irradiance data were acquired and computed to calculate the DNI given the exposure angle of the solar concentrator input aperture. The predicted working current at 2.7 V was then computed scaling the certified curve based on the DNI.

*Indoor Testing*: Indoor experiments utilized simulated sunlight emitted from a 300 W ScienceTech solar simulator (Sciencetech SF300) equipped with an AM 1.5 filter. Incidence angles were also swept using a rotating mirror (Figure 4a). Solar simulator spectral characteristics is provided in Figure S5 (Supporting Information).

*Working Cell Potential Calculation*: The cell working potential, $E_{\mathrm{cell}}^{0}$, was calculated according to Equation (5); the change in Gibbs free energy for the process, $\Delta G_{\mathrm{total}}^{0}$, was calculated according to Equation (6) as the addition of the reaction free energy change and the ideal Gibbs energy of mixing for the species dissolved in the electrolyte

(5) $$E_{\mathrm{cell}}^{0}=-\frac{\Delta G_{\mathrm{total}}^{0}}{nF}$$

(6) $$\Delta G_{\mathrm{total}}^{0}=\Delta G_{\mathrm{reaction}}^{0}+\Delta G_{\mathrm{mixing}}^{0}$$

(7) $$\Delta G_{\mathrm{reaction}}^{0}=\sum_{i}\upsilon_{i}\cdot \Delta G_{i}^{0}(p)-\sum_{j}\upsilon_{j}\cdot \Delta G_{j}^{0}(r)$$

(8) $$\Delta G_{\mathrm{mixing},\mathrm{id}}^{0}=RT\sum_{k}x_{k}\cdot \ln(x_{k}^{})$$

*n* being the number of electrons involved; *F* is the Faraday constant; $\upsilon_{i}$ is the stechiometric coefficient of the specie i; $\Delta G_{i}^{0}$ and $\Delta G_{j}^{0}$ are the standard Gibbs energy changes associated with the specie i (products) or j (reactants); *R* is the universal gas constant; *T* is the operative temperature; *x*_k_ the molar fraction of the specie k. Calculated considering ideal solutions, $\Delta G_{\mathrm{mixing}}^{0}$ contribution was proven to be negligible compared to $\Delta G_{\mathrm{reaction}}^{0}$; $E_{\mathrm{cell}}^{0}=E_{\mathrm{cathode}}^{0}-E_{\mathrm{anode}}^{0}$ was therefore considered as the cell working potential.

## Conflict of Interest

The authors declare no conflict of interest.

## Supporting information

Supplementary

## References

[1] S. Bilgen , S. Keleş , A. Kaygusuz , A. Sarı , K. Kaygusuz , Renewable Sustainable Energy Rev. 2008, 12, 372.

[2] G. G. Botte , Electrochem. Soc. Interface 2014, 23, 49.

[3] J. Jia , L. C. Seitz , J. D. Benck , Y. Huo , Y. Chen , J. W. D. Ng , T. Bilir , J. S. Harris , T. F. Jaramillo , Nat. Commun. 2016, 7, 13237. 27796309 10.1038/ncomms13237 PMC5095559

[4] J.‐W. Schüttauf , M. A. Modestino , E. Chinello , D. Lambelet , A. Delfino , D. Dominé , A. Faes , M. Despeisse , J. Bailat , D. Psaltis , C. Moser , C. Ballif , J. Electrochem. Soc. 2016, 163, F1177.

[5] B. Mei , G. Mul , B. Seger , Adv. Sustainable Syst. 2017, 1, 10.1002/adsu.201600035.

[6] *Chlorine Market: Global Industry Analysis and Opportunity Assessment 2015–2025*, Future Market Insights (FMI), n.d.

[7] J. Chlistunoff , Final Technical Report ‐ Advanced Chlor‐Alkali Technology, LAUR 05‐2444, DOE Award 03EE‐2F/ED190403 Project Period 10/01 – 09/04 2005.

[8] EuroChlor, 2014–2015 Chlorine Industry Review, 2015.

[9] W. Luo , Z. Yang , Z. Li , J. Zhang , J. Liu , Z. Zhao , Z. Wang , S. Yan , T. Yu , Z. Zou , Energy Environ. Sci. 2011, 4, 4046.

[10] E. Worrell , D. Phylipsen , D. Einstein , N. Martin , Energy Use and Energy Intensity of the U.S. Chemical Industry, Energy Analysis Department, Environmental Energy Technology Division, Ernst Orland Lawrence Berkeley National Laboratory, University of California, California, USA 2000.

[11] *Renewable Energy Technologies: Cost Analysis Series – Solar Photovoltaics*, International Renewable Energy Agency (IRENA), n.d.

[12] *International Technology Roadmap for Photovoltaic (ITRPV), 2015 Results, Seventh Edition (March 2016)*, VDMA Photovoltaic Equipment, Germany, n.d.

[13] K. Khouzam , in Conf. Record of the Twenty‐Ninth IEEE Photovoltaic Specialist Conf. 2002, IEEE, New Orleans, LA, USA 2002, pp. 1508–1511.

[14] K. Y. Khouzam , in 2008 IEEE Power Energy Society General Meeting ‐ Conversion and Delivery of Electrical Energy 21st Century, IEEE, Pittsburgh, PA, USA 2008, pp. 1–8.

[15] K. Y. Khouzam , in Proc. 3rd World Conf. on Photovoltaic Energy Conversion 2003, Vol. 3, IEEE, Osaka, Japan 2003, pp. 2443–2446.

[16] J. Appelbaum , K. Y. Khouzam , Y. Dagan , in 2006 IEEE 24th Convention on Electrical and Electronics Engineers in Israel, IEEE, Eilat, Israel 2006, pp. 21–24.

[17] H. Park , C. D. Vecitis , M. R. Hoffmann , J. Phys. Chem. C 2009, 113, 7935. 10.1021/jp807116q 19123849

[18] J. Radjenovic , D. L. Sedlak , Environ. Sci. Technol. 2015, 49, 11292. 26370517 10.1021/acs.est.5b02414

[19] J. M. Barazesh , C. Prasse , D. L. Sedlak , Environ. Sci. Technol. 2016, 50, 10143. 27599127 10.1021/acs.est.6b02232 PMC5032050

[20] T. Takamoto , E. Ikeda , H. Kurita Masamichi Ohmori , Appl. Phys. Lett. 1997, 70, 381.

[21] F. Dimroth , S. Kurtz , MRS Bull. 2007, 32, 230.

[22] M. Woodhouse , A. Goodrich , A Manufacturing Cost Analysis Relevant to Single‐ and Dual‐Junction Photovoltaic Cells Fabricated with III‐Vs and III‐Vs Grown on Czochralski Silicon, The Renewable Energy Laboratory (NREL), Denver, CO, USA 2013.

[23] C. Turchi , Parabolic Trough Reference Plant for Cost Modeling with the Solar Advisor Model (SAM), The Renewable Energy Laboratory (NREL), Denver, CO, USA 2010.

[24] M. R. Shaner , H. A. Atwater , N. S. Lewis , E. W. McFarland , Energy Environ. Sci. 2016, 2354.

[25] J. S. Price , A. J. Grede , B. Wang , M. V. Lipski , B. Fisher , K.‐T. Lee , J. He , G. S. Brulo , X. Ma , S. Burroughs , C. D. Rahn , R. G. Nuzzo , J. A. Rogers , N. C. Giebink , Nat. Energy 2017, 2, 17113.

[26] C. A. Rodriguez , M. A. Modestino , D. Psaltis , C. Moser , Energy Environ. Sci. 2014, 7, 3828.

[27] Design & interactie Fabrique [merken, “National Water Footprint,” can be found under /en/water‐footprint/national‐water‐footprint/, (accessed: July 2017).

[28] M. A. Modestino , D. F. Rivas , S. M. H. Hashemi , J. G. E. Gardeniers , D. Psaltis , Energy Environ. Sci. 2016, 9, 3381.

[29] The SunShot Initiative's 2030 Goal: 3¢ per Kilowatt Hour for Solar Electricity, Department of Energy (DoE), 2016.

[30] H. Mousazadeh , A. Keyhani , A. Javadi , H. Mobli , K. Abrinia , A. Sharifi , Renewable Sustainable Energy Rev. 2009, 13, 1800.

[31] E. J. Tremblay , D. Loterie , C. Moser , Opt. Express 2012, 20, A964. 23326844

[32] V. Zagolla , D. Dominé , E. Tremblay , C. Moser , Opt. Express 2014, 22, A1880. 25607502 10.1364/OE.22.0A1880

[33] D. Bergner , M. Hartmann , J. Appl. Electrochem. 1993, 23, 103.

[34] T. F. O'Brien , T. V. Bommaraju , F. Hine , Handbook of Chlor‐Alkali Technology: Vol. I: Fundamentals, Vol. II: Brine Treatment and Cell Operation, Vol. III: Facility Design and Product Handling, Vol. IV: Operations, Volume V: Corrosion, Environmental Issues, and Future Developments, Springer Science & Business Media, NYC, NY, USA 2007.

[35] P. D. H. Wendt , P. D. G. Kreysa , in Electrochemical Engineering, Springer, Berlin/Heidelberg 1999, pp. 252–289.

[36] R. K. B. Karlsson , A. Cornell , Chem. Rev. 2016, 116, 2982. 26879761 10.1021/acs.chemrev.5b00389

[37] T. Dennis , J. B. Schlager , K. A. Bertness , IEEE J. Photovolt. 2014, 4, 1119.

[38] R. E. Hart , D. J. Brinker , K. A. Emery , in Conf. Record of 20th IEEE Photovoltaic Specialists Conf., Vol. 1, IEEE, Las Vegas, NV, USA 1988, pp. 764–765.

[39] K. Emery , PV Cell and Module Calibrations at NREL, National Renewable Energy Laboratory (NREL), n.d.

[40] MIOX ‐ Blackwater Treatment Capacities, MIOX, 2014.

[41] “EIA: CBECS 2012 Water consumption in large buildings summary,” can be found under https://www.eia.gov/consumption/commercial/reports/2012/water/, (accessed: July 2017).

