# Supplementary material for: A 25.1% Efficient Stand‐Alone Solar Chloralkali Generator Employing a Microtracking Solar Concentrator
Source: Glob Chall. 2017 Nov 29;1(9):1700095. doi: 10.1002/gch2.201700095 (PMC6607182; doi:10.1002/gch2.201700095)
Supplement: Supplementary file 1 — Supplementary [file GCH2-1-1700095-s001.pdf]

Copyright WILEY-VCH Verlag GmbH & Co. KGaA, 69469 Weinheim, Germany, 2017.

Global Challenges
Open Access

Supporting Information

for *Global Challenges,* DOI: 10.1002/gch2.201700095

A 25.1% Efficient Stand-Alone Solar Chloralkali Generator Employing a Microtracking Solar Concentrator

Enrico Chinello, Miguel A. Modestino, Laurent Coulot, Mathieu Ackermann, Florian Gerlich, Demetri Psaltis, and Christophe Moser*

Supplementary Material for

A 25.1% efficient stand-alone solar chlor-alkali generator employing a micro-tracking solar concentrator

Enrico Chinello[a], Miguel A. Modestino[b], Laurent Coulot[c], Mathieu Ackermann[c], Florian Gerlich[c], Demetri Psaltis[a] and Christophe Moser[a*]

[a]: School of Engineering, Ecole Politechnique Federale de Lausanne (EPFL), Switzerland.

[b]: Tandon School of Engineering, New York University (NYU), Brooklyn, NYC, United States.

[c]: Insolight Sarl, Ecublens, Switzerland.

*Corresponding author: Enrico Chinello, enrico.chinello@epfl.ch, tel: +41 21 69 35171

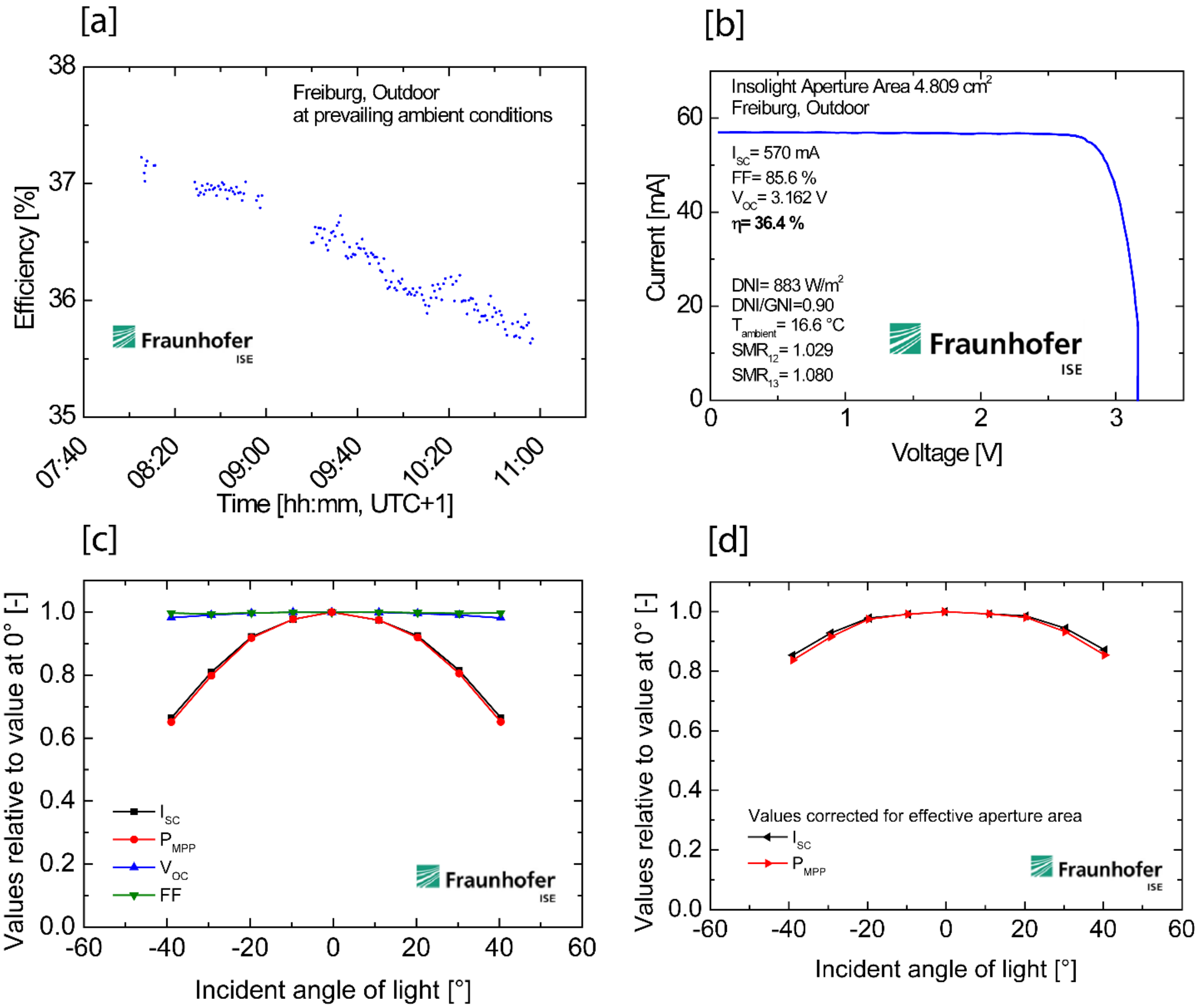

Figure S1 [a]: Overall electrical efficiency of the Semprius cells illuminated with Insolight optical concentrator; values within 36÷37% were recorded. [b]: Reference certified polarization curve of the solar cells, input direct normal irradiance (DNI) 883 W/m^2. [c]: short-circuit current (I_{SC}), power at maximum power point (P_{MPP}), open circuit voltage (V_{OC}) and fill-factor (FF) for difference incidence angles; tests at Fraunhofer ISE demonstrated that our solar concentrator, thanks to its millimetric displacements, is capable of guaranteeing ~60% optical efficiency when the input illumination tilt is 40° with respect to the normal. [d]: P_{MPP} and I_{SC} corrected for the effective input area; we utilized these values for extrapolating the predicted working currents.

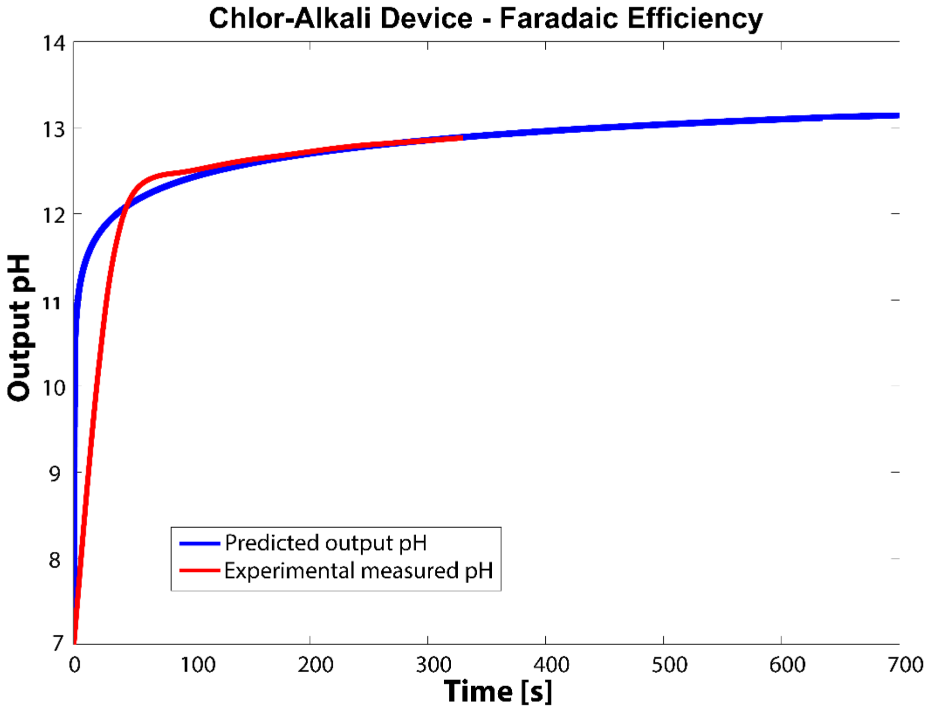

***Figure S2**: Measurement of the Faradaic efficiency of the solar chlor-alkali device.* We tested the electrochemical efficiency measuring the pH of the output catholyte; ions OH^- produced in the process (Equation (3) main manuscript) are in fact in direct correspondence with the evolution of the main products, H_2 and Cl_2. This technique was adopted given the high solubility (~6g/kgwater, 25°C) of chlorine in water, impeding a direct measurement of the evolved gas. Device configuration is described in the "Experimental section" of the main manuscript; current density was externally imposed (200 mA). The beaker containing the catholyte output tube was initially filled with 600 ml of de-ionized water to smooth the pH ramp. The predicted pH curve (blue line) was calculated considering the amount of hydroxyl ions OH^- deriving from the input solution (0.5M NaOH) and the chlor-alkali reaction. The red curve corresponds to the pH values measured experimentally utilizing a VWR pH110 pH-meter, calibrated using Merck Certipur® tampon solutions (pH 4.01/7.00/10.01). After an initial stabilization phase, the two curves show good accordance: the calculated faradaic efficiencies are within 96-99%.

The average current density recorded throughout the outdoor experiment (6.25 mA·cm^{-2} – Figure 3b main text) was considered to calculate the Cl_2 and H_2 production rates, according to Faraday's law.

$$\dot{n}_{Cl_2,H_2} = \frac{I \cdot \Delta t}{z \cdot F} \cdot \quad _{Faraday} = \frac{6.25\ mA \cdot cm^{-2} \cdot 4.809\ cm^2 \cdot 3600\ s}{2 \cdot 96485\ C \cdot mol^{-1}} \cdot 0.97 = 5.44 \cdot 10^{-4}\ mol \cdot hour^{-1} \tag{1}$$

$$\dot{m}_{Cl_2} = \dot{n}_{Cl_2} \cdot M_{m_{Cl_2}} = 38.6\ mg \cdot hour^{-1} \qquad \dot{m}_{H_2} = \dot{n}_{H_2} \cdot M_{m_{H_2}} = 1.09\ mg \cdot hour^{-1} \tag{2}$$

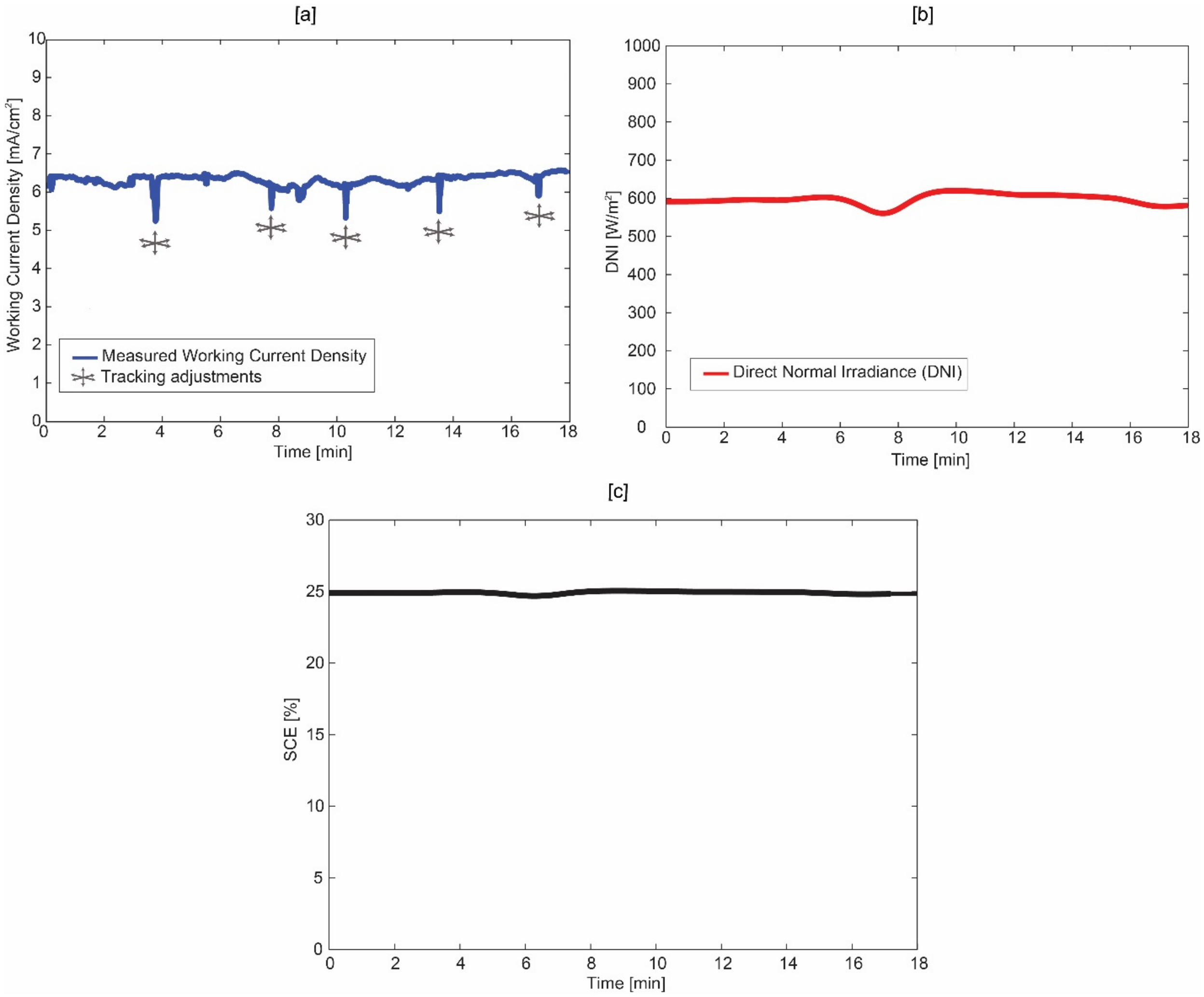

Figure S3 [a]: Experimentally measured current density circulating in the solar chlor-alkali device [b]: Direct Normal Irradiance (DNI) for the experimental period, calculated using the Direct Horizontal Irrandiance (DHI) and the Angle of Incidence (AOI) on the input aperture of the solar concentrator. [c]: resulting Solar-to-Chemical conversion efficiency (SCE); yields calculation is described in the main text.

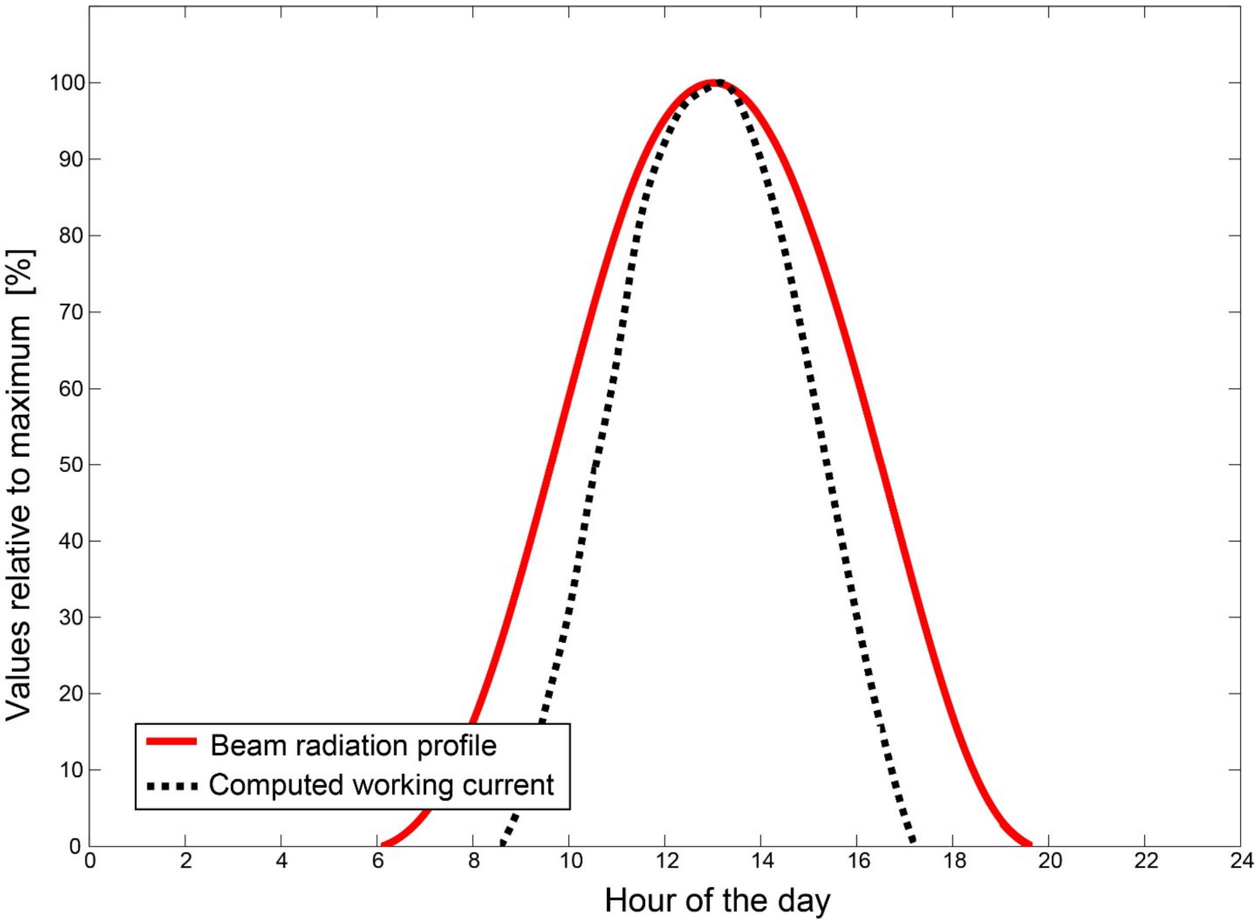

***Figure S4**: Beam irradiance profile and computed working current.* The graph refers to the indoor reconstruction of a typical summer day (21st June, in Lausanne – CH). The red curve displays the solar beam power profile and it was obtained by EnergyPlus weather data (https://energyplus.net/weather), whereas the dashed black line corresponds to the theoretical working current expected to circulate in the chlor-alkali device; the latter considers the optical efficiency of the solar concentrator depicted in Supplementary Fig. 1. The difference between the two curves derives from the optical efficiency of the solar concentrator (Figure S1). The two curves are normalized with respect to the maximum values (i.e. at 13h), 860 mW/cm^2 and 3.6 mA/cm^2, respectively.

Range (nm)	ASTM Standard (%)	Solar Simulator (%)
**400-500**	16.9 %	22.97 %
**500-600**	19.7 %	20.66 %
**600-700**	18.5 %	18.03 %
**700-800**	15.2 %	13.91 %
**800-900**	12.9 %	11.59 %
**900-1100**	16.8 %	12.83 %
**SUM**	100 %	100 %

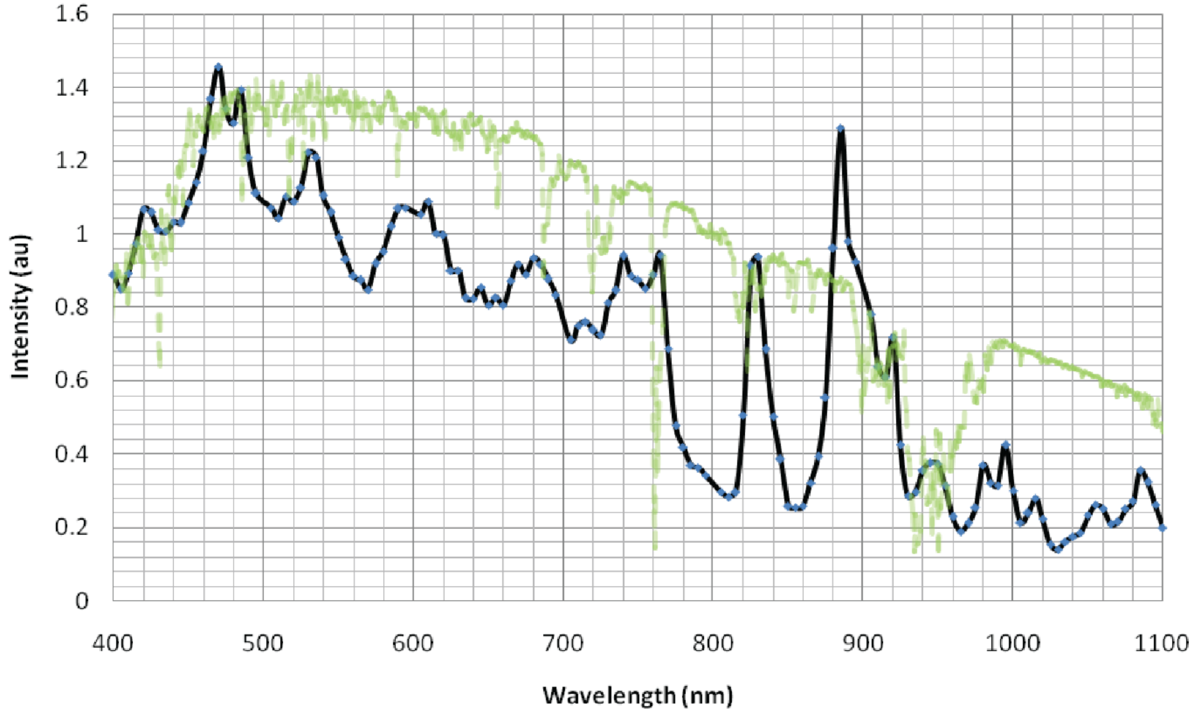

Figure S5: *Sciencetech SF300 solar simulator spectral data*. The ASTM standard for direct normal incidence dictates that for Class A spectral match to be achieved the percentage of power contained in each interval set out by the standard must deviate no more than ±25% from the standard. The results are the measured spectrum of our lens based Sciencetech SF300 solar simulator using a Xenon short-arc lamp. The results of the measurement indicate a Class A spectral match based on the ASTM standard for global irradiation. The provided graph shows the measured spectrum of the solar simulator (black) and the solar reference spectrum (green) for AM1.5G.

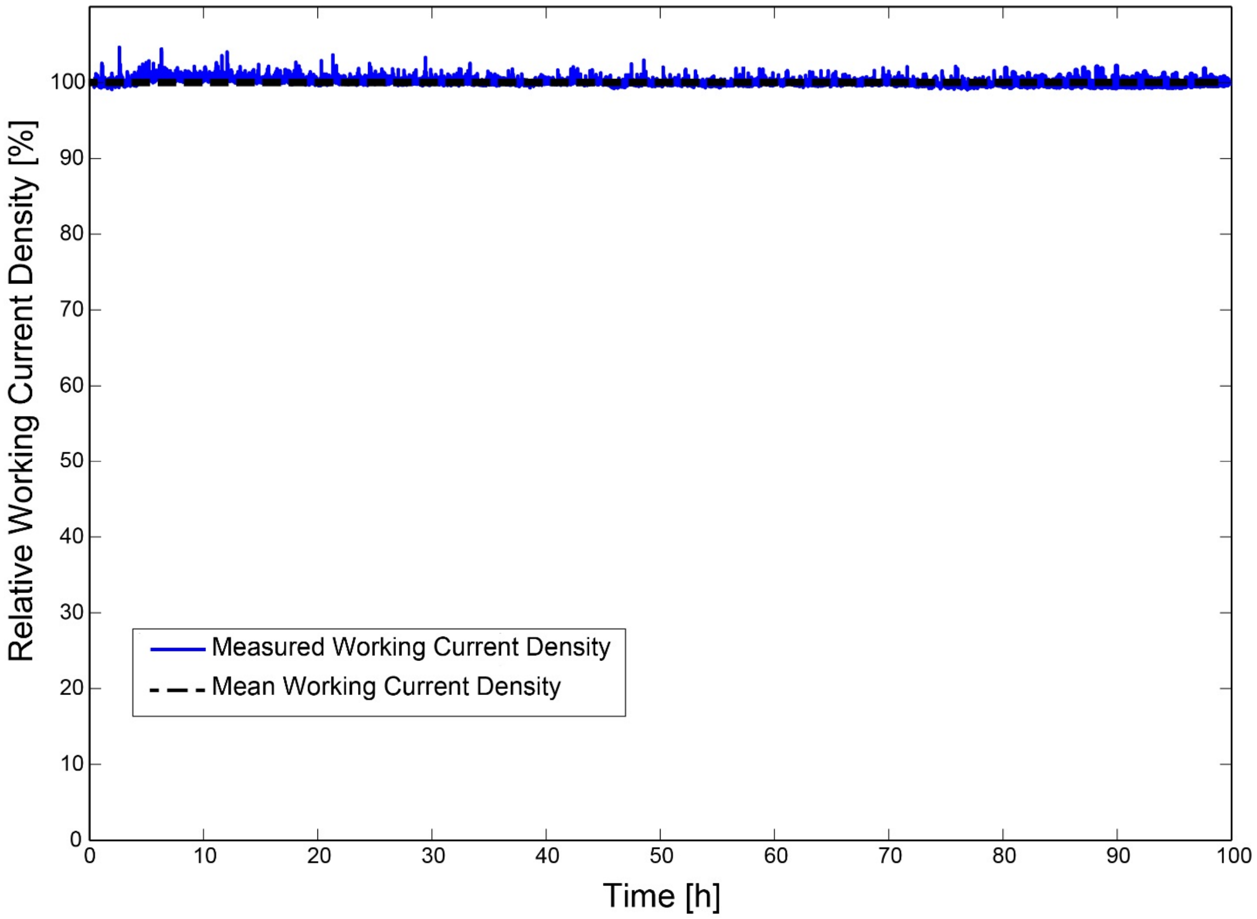

***Figure S6**: Performance stability of the solar chlor-alkali generator over time.* The device was tested in continuous operation for the most severe working conditions recorded in the indoor summer day reconstruction (i.e. data point corresponding to 13h – 3.6 mA·cm^{-2} - Fig. 4b, main text). The setup employed is depicted in Fig.4a (main text) and the experimental conditions are those reported in the "Methods" section. The measured working current density (blue line) is depicted in relative terms with respect to the mean working current density (dashed black line). Little fluctuations were observed and originated from oscillations of the solar simulator beam power (consistently with Figure S6). The standard deviation of the measurement was 1.37%. The experiments demonstrated the capability of operating continuously and stably for more than 100 hours.

Table S1. *Support table for solar-powered chlorine generators technoeconomic analysis.*

**Chassis[1–3]**	7.1	$\$\cdot m^{-2}$
**Semiconductors (GaAs 3 junction)[4]**	175-750	$\$\cdot m^{-2}$
**Catalysts (DSAs)**	200	$\$\cdot m^{-2}$
**Membrane (Nafion)[5]**	5	$\$\cdot m^{-2}$
**Tracker hardware[6]**	44.8	$\$\cdot m^{-2}$
**Concentrator[7]**	24-48	$\$\cdot m^{-2}$
**Piping[8]**	1.6	$\$\cdot m^{-2}$
**Controls[8]**	8.9	$\$\cdot m^{-2}$
**Panel mounting[9]**	29	$\$\cdot m^{-2}$
**Labor[9]**	29	$\$\cdot m^{-2}$
**Other BOS[9]**	59	$\$\cdot m^{-2}$
**Inflaction rate *r***	1.9	%
η_{SCE}	25	%
**Cl_2 production per day**	2.5	$kg\cdot day^{-1}$

$$Capacity\ Factor\ CF = \frac{\sum Solar\ Irradiances\ (hourly)[W\cdot m^{-2}\cdot hour^{-1}]}{1000\ W\cdot m^{-2}\cdot 8760\ hours}$$

$$Area\ A\ [m^2] = \frac{Cl_2\ production\ \left[ton_{Cl_2}.day^{-1}\right]\cdot 2.72\ \left[J\cdot ton_{Cl_2}{}^{-1}\cdot V^{-1}\right]\cdot 2.7\ V}{1000\ [W\cdot m^{-2}]\cdot \eta_{SCE}\cdot 3600\cdot 24\cdot CF}$$

$$Capital\ Expenses = \sum Specific\ Cost\ [\$\cdot m^{-2}]\cdot A\ [m^2]$$

$$Operating\ Cost = \sum_{year=1}^{20} \frac{Replacement\ Costs\ \ [\$\cdot m^{-2}]\cdot A\ [m^2]}{(1+r)^{year}}$$

$$Product\ Revenue = \sum_{year=1}^{20} \frac{Cl_{2,daily}\ \left[ton_{Cl_2}.day^{-1}\right]\cdot 365\cdot LC_{Cl_2}\ [\$\cdot ton^{-1}]}{(1+r)^{year}}$$

$$Net\ Present\ Value\ NPV = 0 = Product\ Revenue - Operating\ Cost - Capital\ Expenses$$

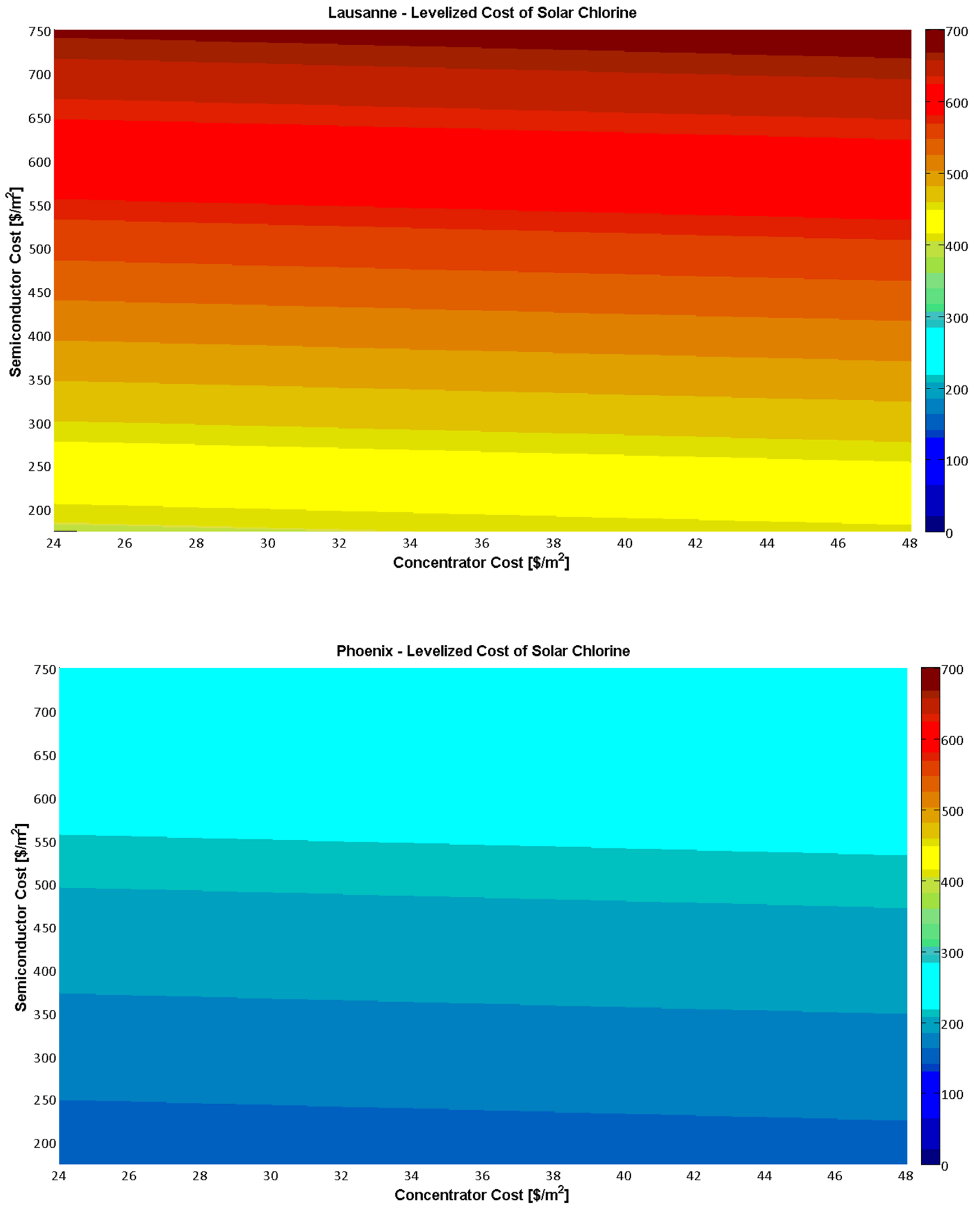

Figure S7*:: Levelized cost of chlorine in Lausanne (CH) and Phoenix (U.S.).* Solar concentrator cost was varied within 24-48 $\$\cdot m^{-2}$, GaAs semiconductor cost was varies within 175-750 $\$\cdot m^{-2}$ *[10,11]*.

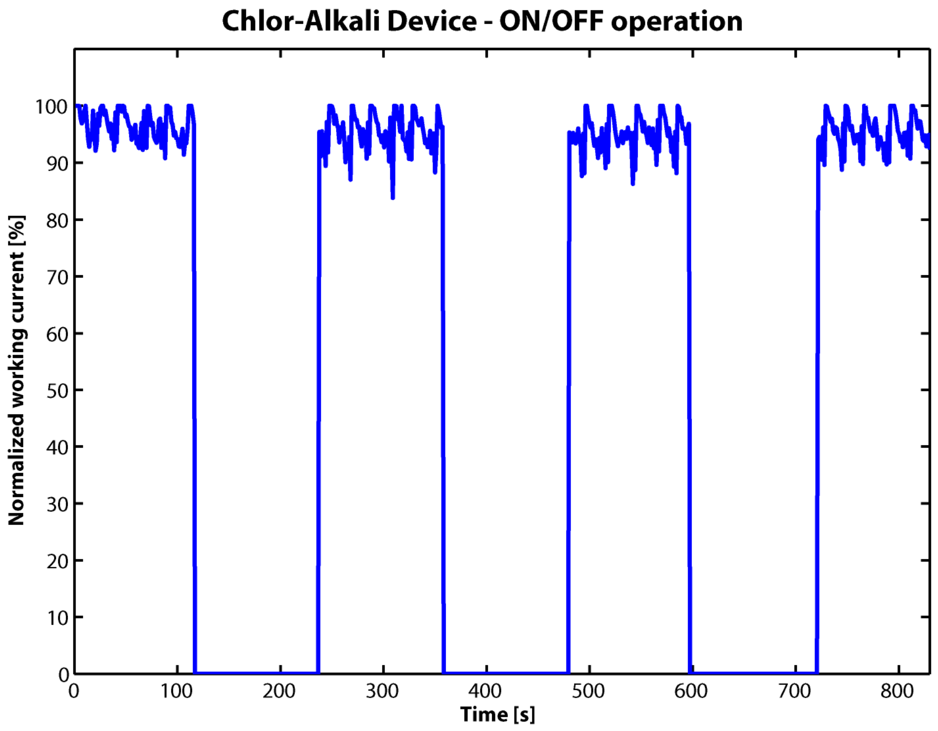

***Figure S8**: ON-OFF operation cycles.* Simulated sunlight (ScienceTech SF300, AM 1.5 filter) was utilized to recreate the alternation of day and night in ON-OFF cycles. Solar cells were alternatively illuminated and shadowed with 120 seconds intervals. Figure indicates that a current circulating inside the device was recorded when the solar cells were illuminated solely. The current is represented in relative percentage terms with respect to the maximum value. Fluctuations in the current level are due to fluctuation in the solar simulator beam intensity.

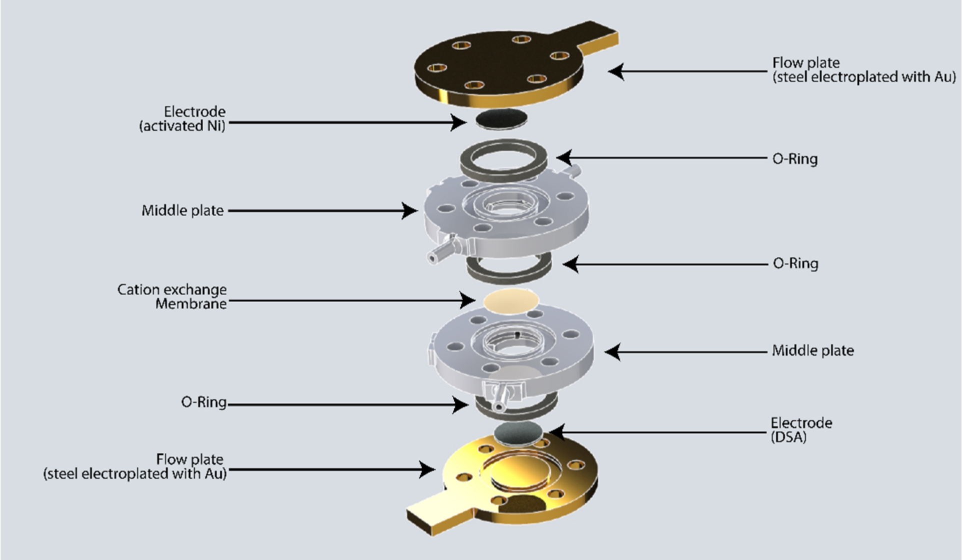

***Figure S9**: Electrolyzer exploded view.* Two flow-plates, fabricated in steel and electroplated with gold by Shapeways Inc (New York, U.S.), conduct the charges; the electroplated gold layer proved higher stability than an alternative sputtered layer. The flow plates provided casing for the electrodes. The anode was a DSA® circular piece (Ø 13 mm), whereas the cathode was a circular piece of activated nickel grid (Ø 13 mm). Electrodes were purchased from *Industrie* De Nora S.p.A. (Milan, Italy). Two intermediate plate were 3D printed using a Stratasys tool at the Additive Manufacturing Atelier at EPFL (Lausanne, Switzerland); these pieces hold the electodes and the middle cation exchange membrane. The membrane was purchased from IonPower and is DuPont™Nafion® PFSA membrane 117. Sealing was guaranteed utilizing NBR 70° O-rings.

Hour of the day	Solar simulator beam power [%]	Incidence Angle [°]	Computed Current [%]	Mean Measured Current [%]	STD Mean Measured Current [%]	SCE [% of peak value]
**9**	27.6	-49.7	16.33	23.35	1.78	59.2
**10**	51.3	-36.1	38.37	35.57	2.98	74.8
**11**	74.8	-22.5	70.17	69.45	3.26	93.8
**12**	95.9	-8.7	93.66	95.02	3.77	97.7
**13**	100.0	5.1	100.00	99.80	3.36	100.0
**14**	98.6	18.8	90.48	88.33	4.06	91.8
**15**	86.3	32.5	65.60	60.63	2.78	76.0
**16**	65.8	46.1	35.05	33.29	2.28	53.3
**17**	42.7	59.6	8.71	14.26	2.16	20.4

***Table S2**: values plotted in Figure 3.* The table reports the computed and the measured current during the indoor test recreating the solar path of a typical summer day. The day chosen to be reconstructed is 21st June (summer solstice), in the location of Lausanne. The summer day was recreated indoor (Fig. 3a main manuscript) using simulated sunlight, tuning the beam power and adjusting the tilt of a rotating mirror according to the values for each hour of the chosen day (9h-17h) according to weather data obtained from Energy Plus (https://energyplus.net/weather). The solar simulator was calibrated in order to have in the conditions corresponding to 13h a beam output power of 883 W/m^2; in these conditions the short circuit current density was 3.6 mA/cm^2. The power levels for the other data points were calculated according to the beam radiation profile provided by EnergyPlus (red line, Supplementary Fig. 5). The values recorded for the other data points were referred to this value. The solar cells were then illuminated for 180 seconds intervals in those conditions to obtain an average value and a standard deviation. The computed values were derived for each hour of the day considering solar illumination direction, intensity and the optical efficiency of our solar concentrator.

In general, the different PV characteristics curves were extrapolated from the certified curve at DNI 883 W/m^2 (Fraunhofer ISE, Freiburg, Germany) using the equation

$$I = I_0 \cdot (e^{\frac{q \cdot V}{n \cdot k \cdot T}} - 1) \quad (3)$$

Where I is the calculated current, I_0 is the dark saturation current, q is the elementary electron charge (1.6·10^{-19} C), V is the considered voltage, n is the ideality factor (considered 1), k is a constant value (1.38·10^{-23} J/K), T is the cell temperature in K.

[1] "Solar Glass Price Plunge to Cease as Trade Sanctions Take Effect | IHS Online Newsroom," can be found under http://news.ihsmarkit.com/press-release/design-supply-chain-media/solar-glass-price-plunge-cease-trade-sanctions-take-effect, **n.d.**
[2] "Injection Molded Plastic Market | Industry Report, 2022," can be found under http://www.grandviewresearch.com/industry-analysis/injection-molded-plastics-market, **n.d.**
[3] "S&P Global Platts Petrochemical Index (PGPI) - August 2017 | Petrochemicals | Platts," can be found under https://www.platts.com/news-feature/2014/petrochemicals/pgpi/polypropylene, **n.d.**
[4] M. Woodhouse, A. Goodrich, *A Manufacturing Cost Analysis Relevant to Single- and Dual-Junction Photovoltaic Cells Fabricated with III-Vs and III-Vs Grown on Czochralski Silicon*, The Renewable Energy Laboratory (NREL), U.S.A., **2013**.
[5] L. Puskar, E. Ritter, U. Schade, M. Yandrasits, S. J. Hamrock, M. Schaberg, E. F. Aziz, *Phys. Chem. Chem. Phys.* **2017**, *19*, 626–635.
[6] "Solar Market Insight Report 2014 Q2," can be found under /research-resources/solar-market-insight-report-2014-q2, **n.d.**
[7] C. Turchi, *Parabolic Trough Reference Plant for Cost Modeling with the Solar Advisor Model (SAM)*, The Renewable Energy Laboratory (NREL), U.S.A., **2010**.
[8] B. A. Pinaud, J. D. Benck, L. C. Seitz, A. J. Forman, Z. Chen, T. G. Deutsch, B. D. James, K. N. Baum, G. N. Baum, S. Ardo, et al., *Energy Environ. Sci.* **2013**, *6*, 1983–2002.
[9] *The SunShot Initiative's 2030 Goal: 3¢ per Kilowatt Hour for Solar Electricity*, Department Of Enegy (DoE), **2016**.
[10] M. R. Shaner, H. A. Atwater, N. S. Lewis, E. W. McFarland, *Energy Env. Sci* **2016**, DOI 10.1039/C5EE02573G.
[11] M. Dumortier, S. Tembhurne, S. Haussener, *Energy Environ. Sci.* **2015**, *8*, 3614–3628.
